# Development of a Cuvette-Based LSPR Sensor Chip Using a Plasmonically Active Transparent Strip

**DOI:** 10.3389/fbioe.2019.00299

**Published:** 2019-11-01

**Authors:** Seo Yeong Oh, Nam Su Heo, Vivek K. Bajpai, Sung-Chan Jang, Gyeongsik Ok, Youngjin Cho, Yun Suk Huh

**Affiliations:** ^1^Department of Biological Engineering, Inha University, Incheon, South Korea; ^2^Division of Electron Microscopic Research, Korea Basic Science Institute, Daejeon, South Korea; ^3^Department of Energy and Materials Engineering, Dongguk University-Seoul, Seoul, South Korea; ^4^Radwaste Management Center, Korea Atomic Energy Research Institute, Daejeon, South Korea; ^5^Research Group of Consumer Safety, Korea Food Research Institute, Wanju-gun, South Korea

**Keywords:** plasmon chip, localized surface plasmon resonance (LSPR), portable sensor chip, self-assembly method, C-reactive protein (CRP)

## Abstract

This research demonstrates the development of a transmission-mode localized surface plasmon resonance (LSPR) sensor chip using a cuvette cell system for the sensitive detection of a biomolecule marker such as C-reactive protein (CRP). In order to develop a highly sensitive LSPR sensor chip, plasmonically active gold nanoparticles (AuNPs) were decorated onto various transparent substrates in the form of a uniform, high-density single layer using a self-assembly process. The transparent substrate surface was modified with amine functional groups via (3-Aminopropyl)triethoxysilane (APTES) treatment, and the ligand concentration and temperature (0.5% APTES at 60°C) were then optimized to control the binding energy with AuNPs. The optimized plasmonically active strip was subsequently prepared by dipping the amine-functionalized substrate into AuNPs for 8 h. The optimized plasmonic strip functionalized with anti-CRP was transformed into a portable LSPR sensor chip by placing it inside a cuvette cell system, and its detection performance was evaluated using CRP as a model sample. The detection limit for CRP using our LSPR sensor chip was 0.01 μg/mL, and the detection dynamic range was 0.01–10 μg/mL with a %CV of <10%, thus confirming its selectivity and good reproducibility. These findings illustrate that the highly sensitive portable LSPR biosensor developed in this study is expected to be widely used in a diverse range of fields such as diagnosis, medical care, environmental monitoring, and food quality control.

## Introduction

A biosensor utilizes a biologically recognized functionalized substrate to detect a target molecule and a detector or reader capable of converting biochemical interactions into physical and/or chemical signals (Sepúlveda et al., 2009). Biosensors have been widely employed in various fields such as food safety, medical diagnosis, and environmental monitoring, and a large volume of past research has focused on improving the specificity, affinity, and selectivity of biosensors for target biomolecules in order to develop portable sensors that are more sensitive and field-ready. In particular, label-free optical biosensors have been actively developed for use in portable sensors due to their simple structure and the convenient implementation of the resulting sensor system. In this vein, with the rapid development of nanotechnology, there has been a growing interest in developing portable localized surface plasmon resonance (LSPR) sensors.

The principle of LSPR detection is the measurement of the change in the refractive index that occurs when incident photons and metal nanoparticles are subject to oscillation (Willets and Duyne, 2007; Anker et al., 2008). This advantageous characteristic allows surface states to be monitored and intermolecular interactions to be analyzed (Li et al., 2018). LSPR sensors based on novel metal nanoparticles have proven to be an effective platform for the detection of biological targets, binding kinetics, and protein conformational changes (Zhao et al., 2006; Anker et al., 2008; Baciu et al., 2008; Hall et al., 2008). Due to potential application possibilities for the detection of various biomolecules based on LSPR, effective plasmonic nanoparticle design and synthesis has been a particular focus of research. Some researchers have attempted to synthesize effective plasmonic particles by employing various metal particles such as Au, Ag, and HgS (Chen and Lu, 2009; Jia et al., 2013; Chen et al., 2015), and other researchers have manipulated the shape of nanoparticles along 0–3 dimensions (e.g., nanoprobes, nanorods, mushrooms, and nanoprisms) in order to enhance plasmonic signals (Shen et al., 2013; Sanders et al., 2014; Chen et al., 2015; Zhang et al., 2016). Other studies have reported the development of sensitive LSPR sensors that utilize different receptor types, including aptamers, antibodies, peptides, and chemicals, in order to optimize the selective binding force with the target molecule (Zhang et al., 2014; Li et al., 2016; Thakur et al., 2017; Lee et al., 2018). Despite this past research on plasmonic nanoparticle synthesis and specific receptor screening (Sefah et al., 2010; Wu et al., 2016b; Ozgul et al., 2019), there have been few reports on the optimal methodology for the deposition of plasmonic nanoparticles (Galush et al., 2009; Hsu et al., 2011; Coskun et al., 2014; Im et al., 2014) and the fabrication of portable substrates that can provide stable and reproducible plasmonic signals.

Previously, we reported fabrication of an aptamer-conjugated portable plasmonic biosensor-based device employing AuNPs for the sensitive detection of bacterial cells of *Salmonella typhimurium* (Oh et al., 2017). However, in this study reported here, we optimized several parameters to fabricate a stable and highly sensitive Cys-protein G-functionalized plasmonic substrate conjugated with anti-CRP for using in a portable cuvette-based LSPR sensor chip that can be used in the field diagnosis within a simple transmission-mode optical system for the detection of CRP blood plasma biomarker. Since plasmon absorption signals are determined by the interaction between the substrate on which the gold nanoparticles are deposited and the incident light, the sensor chip signals are affected by the material type and the thickness of the transparent substrate. With this in mind, a total of eight sensor chip substrates, such as polycarbonate (PC) film, 0.4 mm ordinary glass, 0.5 mm ordinary glass, 0.4 mm tempered glass, 0.5 mm tempered glass, 0.5 mm chemical strengthened glass, slide glass, and cover glass were employed and optimization experiments were carried out to determine the optimal plasmonically active substrate for the LSPR sensor chip. The optimized plasmonic substrate was employed in the assembly of the portable LSPR sensor chip by immobilizing it in a disposable plastic cuvette cell system.

**Scheme 1:**
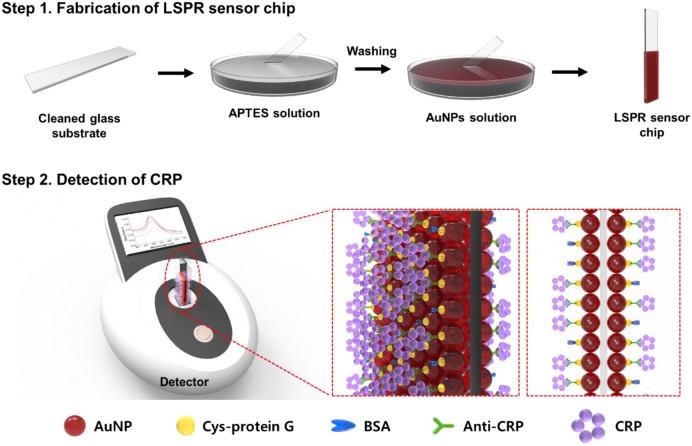
Schematic illustration of the anti-CRP-based LSPR sensor chip for CRP detection.

In order to verify the performance of the fabricated LSPR sensor chip, C-reactive protein (CRP), which is a biomarker for cardiovascular disease and inflammation (Lagrand Wim et al., 1999; Albrecht et al., 2008; Pultar et al., 2009; Bryan et al., 2013), was selected as a model sample and the detection characteristics of the plasmonic substrate were evaluated. We thus established a method of uniformly depositing gold nanoparticles (AuNPs) on a transparent substrate in a single layer using a self-assembled method and placing the substrate in a cuvette cell system to easily fabricate a portable LSPR sensor chip. Our proposed sensor chip produced sensitive detection signals for the target CRP sample, confirming that it can be used in field-based diagnostic detection for a number of applications (Scheme 1).

## Materials and Methods

### Reagents and Apparatus

Gold(III) chloride trihydrate ≥99.9% was purchased from Sigma-Aldrich (St. Louis, MO, USA). Trisodium citrate dehydrate was purchased from Kanto Chemical Co., Inc. (Japan). (3-Aminopropyl) triethoxysilane (APTES) ≥98.0%, bovine serum albumin (BSA) hemoglobin (Hb), transferrin (TRF), and human serum albumin (HSA) were purchased from Sigma–Aldrich. 99.5% methyl alcohol was purchased from Samchun Pure Chemical Co., Ltd. (Korea). All glass substrates were obtained from CARA Nano Glass Technology (Korea). Cysteine-protein G was purchased from ProSpec-Tany TechnoGene Ltd. (USA). Anti-C reactive protein and C-reactive protein were acquired from Bore Da Biotech Co., Ltd. (Korea). Phosphate buffered saline (PBS pH 7.4) was prepared using 0.01 M Na_2_HPO_4_ and 0.01 M NaH_2_PO_4_. Transmission electron microscopy (TEM) (JEM2100F, JEOL Ltd., USA) was used to analyze the structure of the AuNPs. Field emission scanning electron microscopy (SEM) (FE-SEM, S-4300, Hitachi, Japan) was used to analyze the structure of the AuNPs and the substrate surface, and a UV-vis spectrophotometer (V-770, Jasco International Co., Ltd., Japan) was used for plasmon absorption analysis.

### Synthesis of AuNPs

**Figure 1:**
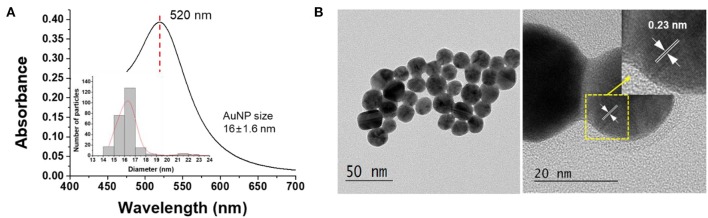
**(A)** UV-vis spectra and **(B)** TEM image of the synthesized AuNPs.

AuNPs were synthesized following the synthesis method previously reported (Oh et al., 2017) for the production of plasmonic substrates. First, 150 mL of 2.2 mM sodium citrate solution was heated to 100°C under rapid stirring for 15 min. When the solution began to boil, 1 mL of 25 mM HAuCl_4_ was added, and 3 mL of the solution was removed after 10 min. After Au seed synthesis, the temperature was lowered to 90°C. After adding 1 mL of 25 mM HAuCl_4_ and stirring for 30 min, 55 mL of the solution was removed, and 53 mL of distilled water and 2 mL of 60 mM sodium citrate solution were added to the remaining solution, followed by stirring for 20 min. After this, 1 mL of 25 mM HAuCl_4_ was added, and the mixture was stirred for 30 min. Another 1 mL of 25 mM HAuCl_4_ was added and the mixture stirred again for 30 min to produce AuNPs of a predetermined size (Figure 1, Figure S1).

### Fabrication of Cuvette-Type LSPR Sensor Chip

#### Development of Plasmonically Active Strips With Various Transparent Substrates

In order to determine the optimal substrate for LSPR sensor chip fabrication, various glass substrates (0.4 mm ordinary glass, 0.5 mm ordinary glass, 0.4 mm tempered glass, 0.5 mm tempered glass, 0.5 mm chemically strengthened glass, slide glass, and cover glass) were coated with AuNPs and the plasmon absorbance was then measured and analyzed. Each glass substrate was soaked in methanol and ultrasonic cleaned for 20 min to remove dust and foreign substances on the glass substrate, followed by washing three times with distilled water to remove the remaining methanol. The glass substrate was subsequently immersed in a 0.5% APTES solution and reacted at 60°C for 1 h. Any APTES that had not bound to the glass substrate was rinsed off with distilled water, and the glass substrate was immersed in the AuNPs solution for 16 h (Figures S2, S3). The glass substrate was then rinsed with water and the absorbance measured using a UV-vis spectrophotometer.

#### Optimization of APTES Concentration on the Plasmonically Active Strips

To analyze the effect of APTES concentration on the detection ability of plasmonically active strips, the glass substrate was first immersed in methanol and sonicated for 20 min to clean it, and the remaining methanol was removed by washing the substrate with distilled water three times. The glass substrate was then immersed in various concentrations of APTES solution (0, 0.0001, 0.0005, 0.001, 0.005, 0.01, 0.05, 0.1, 0.5, 1, and 2%) and reacted at 60°C for 1 h. Any APTES not bound to the glass substrate was removed and the substrate was then immersed in the AuNP solution for 16 h. The absorbance of the glass substrates fabricated using different concentrations of APTES was measured using a UV-vis spectrophotometer.

#### Optimization of APTES Reaction Temperature and Dipping Time for Gold Nanoparticles Solution

After the optimal concentration of APTES had been determined, the deposition efficiency and plasmonic properties of the AuNPs on the substrate were evaluated according to reaction temperature and time. The glass substrate was immersed in an APTES solution prepared at a concentration of 0.5%, and the AuNP deposition efficiency was analyzed for the temperature range 30–70°C at intervals of 10°C. After the ideal APTES functionalization conditions had been determined for the glass substrate (0.5% APTES at 60°C for 1 h), additional experiments were performed to determine the optimal self-assembly reaction time (2, 4, 6, 8, or 10 h) for the deposition of a high density, single layer of AuNPs with amine functional groups on the substrate.

#### pH Test for Stabile Interaction of Anti-CRP on the Plasmonically Active Strips

Plasmonically active strips prepared in this study were reacted for 15 min in PBS buffer solution at different pH values (pH 6, 6.8, 7.4, 7.8, 8.5, 9.5) and the plasmon absorption spectra were confirmed by UV-vis spectrophotometer. In order to determine the optimum active pH condition for anti-CRP, the chip was reacted under the same conditions as above in PBS buffer of various pH including anti-CRP (10 μg/mL), and the difference in the absorption plasmon spectrum depending on the reaction between the plasmonically active strip and anti-CRP was evaluated (Meyer et al., 2007).

### CRP Detection Assay Using a LSPR Sensor Chip

#### Optimization of Cysteine-Protein G and Antibody Binding Affinity Assays

In order to evaluate the detection performance of the fabricated LSPR sensor chip, surface functionalization was analyzed in the detection of the antigen-antibody reaction for the target molecule CRP. For the stable immobilization of the CRP antibody, cysteine-protein G (Cys-protein G) was diluted in PBS solution at three concentrations (1, 10, and 100 μg/mL). For each of the Cys-protein G solutions, 500 μL was added to the LSPR chip and reacted at room temperature for 10 min. To remove unbound Cys-protein G, the LSPR sensor chip was washed twice with PBS and then scanned from 400 to 700 nm with a UV-vis spectrophotometer.

#### CRP Detection Using LSPR Sensor Chip Formats

A 10 μg/mL Cys-protein G-functionalized LSPR chip was reacted with various concentrations (0, 0.01, 0.1, 1, 10, and 100 μg/mL) of anti-CRP (anti-C reactive protein) at room temperature for 10 min and washed twice with PBS. The reaction was blocked using 1% BSA for 30 min to remove noise due to the non-specific binding of the sample, and scanned from 400 to 700 nm with a UV-vis spectrophotometer. The CRP samples were prepared at various concentrations (0, 0.001, 0.01, 0.1, 1, 10, and 100 μg/mL), and the detection performance was evaluated using the antibody-functionalized LSPR sensor chip.

#### Specific Detection of CRP

To confirm the interference effect of the antibody-functionalized LSPR sensor, sample solutions of different proteins and/or antibodies, such as Hb, TRF, HSA, CRP, and mixed sample conditions (Hb+TRF+HSA+CRP) were prepared at a concentration of 0.1 μg/mL. The LSPR sensor chip was reacted for 15 min in a sample solution prepared by various combinations of interference samples, and the selective binding was measured with a UV-vis spectrometer.

## Results and Discussion

### Fabrication of Plasmonically Active Strips for a Cuvette-Based LSPR Sensor Chip

#### Plasmon Absorption Characteristics of Various Transparent Substrates

In this study, we developed a portable LSPR sensor chip with strong potential as a field detection device by optimizing the binding characteristics between plasmonic AuNPs and a transparent substrate. In a transmission-mode LSPR sensor chip, the plasmonic substrate on which AuNPs are deposited efficiently produces resonance phenomena depending on the incident light and the physical reaction conditions, such as the material type and thickness of the substrate, thus generating changes in the plasmon absorption spectrum that can act as a detection signal.

The characteristics of AuNPs synthesized in this study were analyzed by UV-vis spectrophotometer and TEM. Figure 1A shows the absorption spectra of the prepared AuNPs. The synthesized AuNPs showed an intrinsic absorption spectrum (λ_max_) at 520 nm (Ghosh and Chattopadhyay, 2013), and it was confirmed by TEM analysis that AuNPs with a size of 16 ± 1.16 nm were synthesized. As can be seen in Figure 1B, the lattice spacing was 0.23 nm, which corresponds to the (111) plane of the gold cubic phase, indicating that the AuNPs were synthesized as expected (Islam et al., 2015).

**Figure 2:**
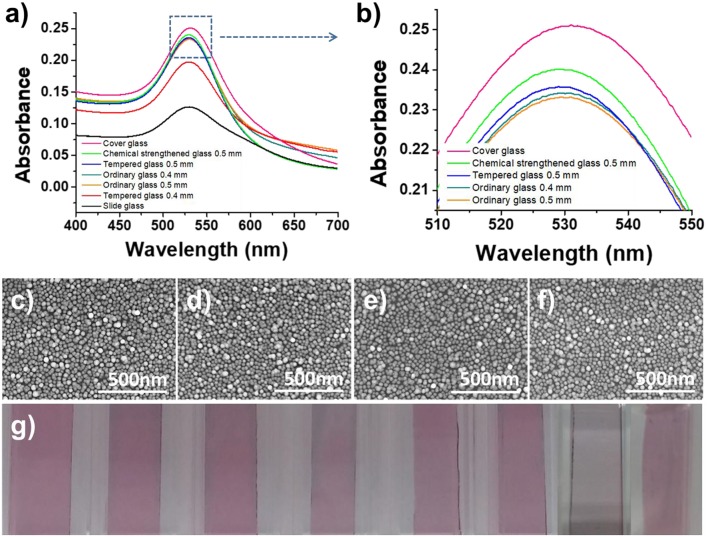
Optimization of the LSPR sensor chip. **(a,b)** Optimization of the glass substrate. **(c–f)** SEM images of immobilized gold nanoparticles on 0.5 mm ordinary glass, 0.5 mm tempered glass, 0.5 mm chemical strengthened glass, and cover glass. **(g)** Optical image of AuNP-coated glass (from left, PC film, 0.4 mm ordinary glass, 0.5 mm ordinary glass, 0.4 mm tempered glass, 0.5 mm tempered glass, 0.5 mm chemical strengthened glass, slide glass, and cover glass).

In this study, transmission-mode LSPR sensor chips were fabricated by depositing AuNPs on a plastic or glass substrate. In order to select the most suitable transparent substrate for the LSPR sensor chip, seven types of glass substrate were employed that differed in either material type or thickness (0.4 mm ordinary glass, 0.5 mm ordinary glass, 0.4 mm tempered glass, 0.5 mm tempered glass, chemically 0.5 mm strengthened glass, slide glass, and cover glass). The plasmonic nanoparticles were deposited at a high density using a self-assembly process between the amine functional groups of the APTES-immobilized glass substrate and the AuNPs. As shown by the SEM images in Figure 2, the AuNPs were uniformly and densely coated on the PC film and all of the glass substrate types.

To evaluate the feasibility of using a transparent substrate deposited with AuNPs as an LSPR sensor chip, the plasmon absorption spectra were compared using a UV-vis spectrometer with a scanning range of 400–700 nm. To ensure the high reproducibility of absorbance measurements and to enable the fabrication of an on-site sensor, each transparent substrate was inserted into a disposable plastic cuvette cell (1 mL) for support to produce a portable sensor chip. In this way, it was possible to fabricate a sensor chip with a high-density layer of AuNPs on all of the transparent substrates under optimal conditions, but the plasmon absorption characteristics differed for each substrate. The thin-walled flexible PC film exhibited the weakest absorption characteristics (data not shown), which was ascribed to the scattering of light due to microscopic bends and the loss of light due to its lower planarity compared to the glass substrates. It was confirmed that the thinner the glass substrate, the higher the absorbance (Figures 2a,b). However, the cover glass, which has the highest absorbance, had low durability because it was too thin, and succeeded in making the portable LSPR sensor chip <50% in the process of assembling the cuvette-based sensor chip. In contrast, with its excellent plasmon absorption characteristics and high fabrication success rate of over 99%, 0.5 mm chemical strengthened tempered glass was selected as the optimal substrate for the proposed portable sensor.

#### Effect of APTES Ligand Concentration on the Fabrication of Plasmonically Active Strips

Based on the previous experimental results, the chemically tempered 0.5 mm glass substrate was used as the transparent strip in the proposed LSPR sensor chip. Several sets of experiments were then conducted to optimize the plasmonically active substrate through the deposition of AuNPs. In the first step, experiments were performed to optimize the immobilization of amine functional groups on the transparent substrate by varying the concentration of APTES, which serves as a linking molecule between the glass strip and the AuNPs. APTES is an aminosilane, which is a linker that plays an important role in the silane bonding with the OH-group of the glass substrate and the amine group bonding with the nanoparticles so that the nanoparticles can be uniformly coated on the glass substrate (Agnihotri et al., 2013). The self-assembly reaction proceeds through electrostatic interactions between positively charged amine groups of functionalized APTES on the glass substrate and negatively charged AuNPs (Nath and Chilkoti, 2004). To determine the optimal concentration of APTES, concentrations of 0, 0.0001, 0.0005, 0.001, 0.005, 0.01, 0.05, 0.1, 0.5, 1, and 2% were tested. After modifying the substrate with the amine functional groups, the plasmon absorption spectra of the AuNP-coated LSPR chip were measured.

**Figure 3:**
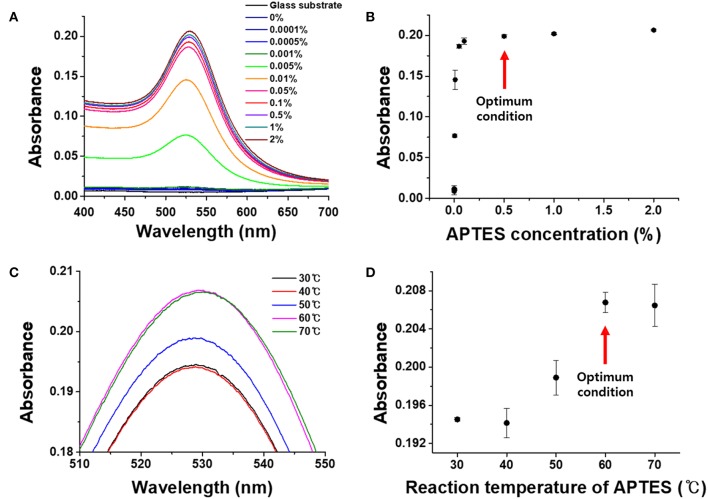
Optimization of APTES conditions on the chemically strengthened glass. **(A)** UV-vis spectra by APTES concentration. **(B)** Absorbance by APTES concentration. **(C)** UV-vis spectra by reaction temperature. **(D)** Absorbance by reaction temperature.

**Figure 4:**
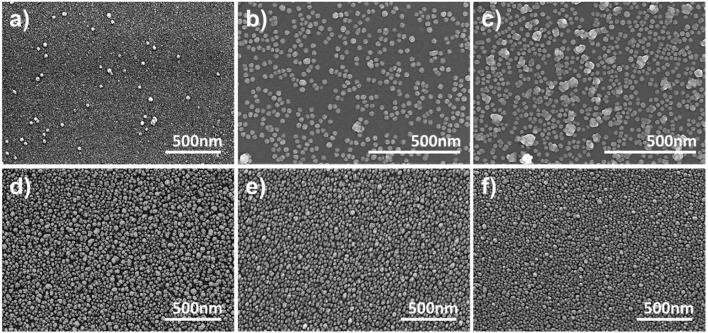
SEM images of immobilized AuNPs on chemically strengthened glass for APTES concentrations of **(a)** 0%, **(b)** 0.005%, **(c)** 0.01%, **(d)** 0.05%, **(e)** 0.1%, and **(f)** 0.5%.

As shown in Figures 3A,B, the absorbance of the LSPR sensor chip for APTES concentrations up to 0.001% was 0.0117, which was not significantly different from the absorbance of a blank glass substrate (0.0051). The absorbance started to gradually increase at an APTES concentration of 0.005%, with the sharpest increase at a concentration of 0.1% (0.1866). There was only a slight increase at a concentration of 0.5% concentration, followed by an almost unchanged absorbance up until a concentration of 2%. The SEM images in Figure 4 correspond with these plasmon absorption spectra results. As expected, only a few AuNPs were observed on the glass substrate with 0.05% APTES, while the AuNPs were deposited most uniformly and densely at a concentration of 0.5%. Based on these experimental results, 0.5% APTES was selected as the optimal concentration for the proposed sensor chip.

#### Effect of APTES Reaction Temperature on the Plasmonically Active Strips

It was confirmed that the degree of immobilization of the AuNPs on the glass substrate was affected by the APTES reaction temperature. To determine the optimal reaction temperature for the binding and immobilization efficiency of APTES, experiments were conducted for temperatures from 30 to 70°C. As can be seen in Figures 3C,D, the plasmon absorbance continued to increase from 30 to 60°C, reaching an absorbance of 0.208. At 70°C, however, the absorbance decreased slightly to 0.207, with a large standard deviation. This is because the APTES was sufficiently functionalized on the substrate surface at 60°C, and raising the temperature to 70°C led to interference and the aggregation of AuNPs due to steric hindrance between the high-density amine functional groups. Therefore, the optimal APTES reaction temperature for the fabrication of the LSPR sensor chip was set at 60°C.

#### Effect of AuNP Dipping Time on the Plasmonically Active Strips

**Figure 5:**
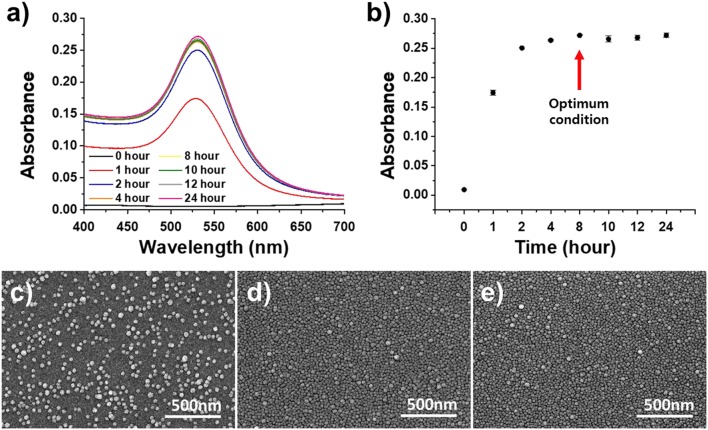
Optimization of the dipping time of the APTES-immobilized glass substrate in the AuNP solution. **(a)** UV-vis spectrum of dipping time. **(b)** Absorbance of dipping time. **(c**–**e)** SEM images for different dipping times (from left: 2, 4, and 6 h).

The stable deposition of the AuNPs on the substrate was analyzed by dipping the functionalized glass substrate into the AuNP solution for different lengths of time. The amine functional groups on the glass substrate and AuNPs underwent a self-assembly reaction to produce a uniform, single layer of AuNPs on the plasmonically active strip, which was then used to fabricate a cuvette-based LSPR sensor in the same manner as described above and assessed with absorbance spectroscopy and SEM (Figure 5). As shown in the Figures 5c–e, the absorbance increased rapidly up to a dipping time of 4 h, before reaching an absorbance of 0.271 after 8 h. The SEM analysis revealed that AuNPs were completely deposited on the surface of the glass substrate after 8 h. Therefore, the optimal dipping reaction time was determined to be 8 h.

#### Effect of pH on the Plasmonically Active Strips

**Figure 6:**
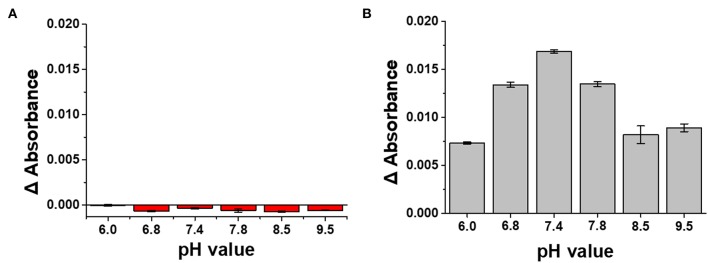
**(A)** Stability test of LSPR sensor chip under various pH conditions. **(B)** Determination of pH condition in PBS buffer for optimizing antibody reaction of LSPR sensor chip.

The stability of the LSPR sensor prepared in this study was evaluated in PBS solution with various pH values ranging from pH 6.0–9.5 (Meyer et al., 2007). As shown in Figure 6A, when the absorbance value of LSPR sensor was measured in 0.1 M PBS buffer at various pH conditions, the change in absorbance value was the smallest at pH 6. In addition, even at pH 6.8, which showed the greatest change, the change of absorbance at the fourth decimal place (−0.0006) occurred only marginally, confirming that the LSPR sensor developed in this study has excellent stability against pH. In addition, when the absorbance value was measured by reacting the plasmonically active chip with anti-CRP (10 μg/mL) in PBS solution of different pH (Figure 6B), it was confirmed that the highest binding force was provided in PBS buffer at pH 7.4.

### Selective CRP Detection Using the Antibody-Functionalized LSPR Sensor

#### CRP Detection Using an Antibody-Functionalized LSPR Sensor Chip

**Figure 7:**
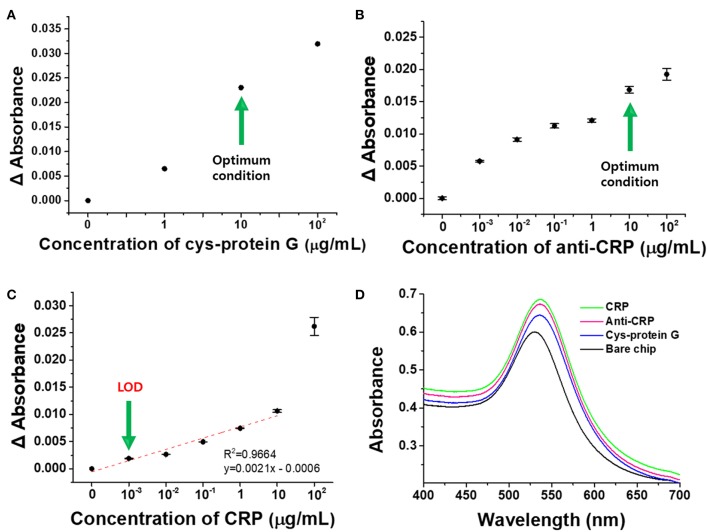
Detection of CRP by anti-CRP-based LSPR chip. **(A)** Optimal Cys-protein G concentration. **(B)** Optimal anti-CRP concentration. **(C)** Detection of CRP at different concentrations. **(D)** Schematic diagram representing the detection of CRP using the anti-CRP-based LSPR sensor chip. All experiments were conducted at least in six measurements, and data represent the mean ± standard deviation. The coefficient of variation (%CV) was below 10%.

In order to evaluate the detection characteristics of the transmission-mode LSPR sensor chip developed in this study, CRP was selected as a model sample and detection experiments were conducted based on the antigen-antibody reaction (Figure 7D). Cys-protein G was immobilized on the fabricated plasmonic strip in order to functionalize antibodies capable of selectively capturing CRP. To determine the appropriate ligand concentration, various concentrations of Cys-protein G (0, 1, 10, and 100 μg/mL) were reacted with the LSPR chip and the change in absorbance was monitored. As expected, the absorbance increased as the concentration of the ligand increased, and 10 μg/mL, which produced the sharpest increase in absorbance, was determined to be the optimal concentration (Figure 7A).

Prior to the binding reaction between the ligand-functionalized chip and the antibody, blocking with 1% BSA was carried out for 30 min to prevent the non-specific reaction of the antibody, and experiments were then performed to optimize the antibody concentration for a range of 0–100 μg/mL (Figure 7B). Cys-protein G and anti-CRP bind to each other by electrostatic and hydrophobic interactions between the protein G and Fc (Kato et al., 1995), so that anti-CRP can be stably functionalized on the LSPR sensor chip and simultaneously detect CRP. As shown in Figure 7B, the change in absorbance was the greatest in the region where the antibody concentration increased from 1 to 10 μg/mL, so the antibody concentration of 10 μg/mL was determined to be the optimal concentration for the detection of CRP in this study (Kim et al., 2013).

The optimized cuvette-based LSPR sensor chip was functionalized with the antibody and its detection performance was then monitored for different concentrations of CRP from 0 to 100 μg/mL. As shown in Figure 7C, the detection limit (LOD) for CRP using the proposed LSPR sensor chip was 11.28 ng/mL whereas the detection range was 0.001–10 μg/mL. Within this dynamic range, the linearity was 0.9964 and the reproducibility was high for 6 or more repeated experiments, and the limit of quantitation (LOQ) was 34.20 ng/mL. LOD and LOQ were calculated from the equations given below (Robouch et al., 2016):

(1) $$\begin{array}{l} x_{LOD}=3.9\times \frac{s_{y}}{b} \end{array}$$

(2) $$\begin{array}{l} \text{LOQ}=3.3\times x_{LOD} \end{array}$$

*x*_*LOD*_ is Limit of detection, *s*_*y*_ is Standard deviation, and b is Slope of the calibration curve.

#### Specific Binding of CRP Using the Antibody-Functionalized LSPR Sensor

**Figure 8:**
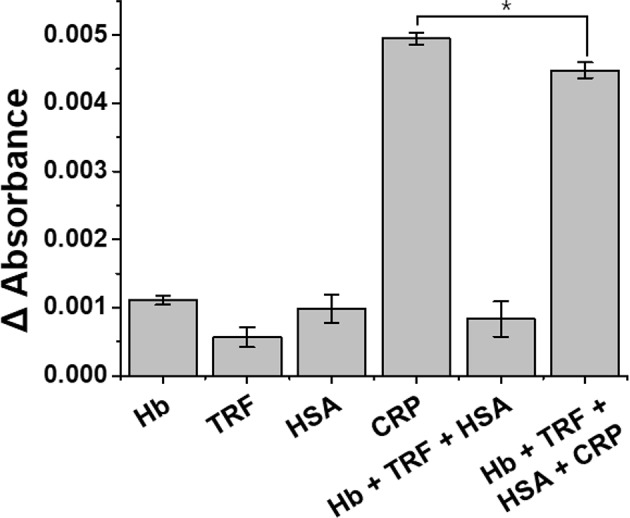
Specificity of anti-CRP functionalized LSPR sensor for the detection of CRP. The selective detection and cross-reactivity to CRP were performed from 0.1 μg/mL complex mixture of proteins (Hb+TRF+HSA+CRP). All experiments were conducted at least in six measurements, and data represent the mean ± standard deviation. The coefficient of variation (%CV) was below 10%, and ^*^LSPR sensitivity was statistically significant for CRP at *p* < 0.01.

In order to evaluate the interference effect from the mixed sample (Hb+TRF+HSA+CRP) with the anti-CRP functionalized LSPR sensor, selective detection, and cross-reactivity to CRP were performed (Figure 8; Ji et al., 2016; Wu et al., 2016a). Our LSPR sensor showed a slight decrease in absorbance of CRP from 0.1 μg/mL concentration of mixed sample solution (Hb+TRF+HSA+CRP) compared to CRP single control sample, but no specificity for other proteins. LSPR sensor was statistically verified for CRP selectivity (*p* < 0.01) and coefficient of variation (%CV) was calculated to confirm its reproducibility. All the experiments were repeated six times under the same condition. The CV should not exceed 20% when performing 10 sample replicates (Reed et al., 2002), and the CV of LSPR sensor yielded a satisfactory precision of <10% (Venosa et al., 2002). Based on these results, we verified that our LSPR sensor can selectively detect CRP under mixed sample conditions.

## Conclusions

In this study, we optimized the fabrication of plasmonically active strips using various transparent substrates and developed a cuvette-based LSPR sensor chip for possible field-ready applications such as point-of-care testing. AuNPs were uniformly deposited at a high density under various ligand concentrations and reaction times on the substrates. Using plasmon absorption spectrum analysis, chemically tempered glass was selected as the optimal substrate for the transmission-mode LSPR sensor chip, with 0.5% APTES functionalized on the glass substrate at 60°C for 1 h. Subsequently, AuNPs were uniformly deposited onto the substrate surface via dipping for 8 h to produce the final plasmonically active strip. A portable transmission-mode antibody-functionalized LSPR chip was then produced by immobilizing the plasmonic strip within a disposable cuvette cell. The detection performance of the LSPR sensor chip developed in this study was evaluated using CRP as a model sample. Based on the antigen-antibody reaction, changes in the plasmon absorption spectrum of the LSPR sensor were measured to detect CRP. After 10 μg/mL Cys-protein G had been bound to the plasmonic strip, it was functionalized with an antibody at 10 μg/mL. The detection sensitivity of the sensor chip was evaluated for various CRP concentrations, with the low detection limit of 0.001 μg/mL confirming the superiority of the detection platform developed in this study. The LSPR chip detection platform developed in this study thus offers a new direction for portable sensors that can be used in various fields such as medical diagnosis, environmental monitoring, and food safety.

## Data Availability Statement

All datasets generated for this study are included in the article/Supplementary Material.

## Author Contributions

The manuscript has been written and composed by SO and NH with the assistance of all other authors. Materials preparation and all experimental work were performed by SO, NH, S-CJ, and GO under guidance of YC and YH. VB and SO revised the manuscript. Data processing was performed by SO, NH, S-CJ, and GO based on the experiments. All authors reviewed the manuscript.

### Conflict of Interest

The authors declare that the research was conducted in the absence of any commercial or financial relationships that could be construed as a potential conflict of interest.
