# Supplementary material for: Development of a Cuvette-Based LSPR Sensor Chip Using a Plasmonically Active Transparent Strip
Source: Front Bioeng Biotechnol. 2019 Nov 1;7:299. doi: 10.3389/fbioe.2019.00299 (PMC6839135; doi:10.3389/fbioe.2019.00299)
Supplement: Supplementary file 1 [file Data_Sheet_1.pdf]

Supporting Information

Development of a Cuvette-Based LSPR Sensor Chip using a Plasmonically Active Transparent Strip

Seo Yeong Oh[1,†], Nam Su Heo[1,2,†], Vivek K. Bajpai[3], Sung-Chan Jang[1, 4], Gyeongsik Ok[5], Youngjin Cho[5,]*, and Yun Suk Huh[1,]*

[1] *Department of Biological Engineering, Inha University, Incheon 22212, Republic of Korea*

[2] *Division of Electron Microscopic Research, Korea Basic Science Institute, Daejeon 34133, Republic of Korea*

[3] *Department of Energy and Materials Engineering, Dongguk University-Seoul, 30 Pildong-ro 1-gil, Seoul, 04620, Republic of Korea*

[4] *Korea Atomic Energy Research Institute, 111, Daedeok-daero 989 beon-gil, Yuseong-gu, Daejeon, 34057, Korea*

[5] *Research Group of Consumer Safety/Research Division of Strategic Food Technology, Wanju-gun, Jeollabuk-do 55365, Republic of Korea*

[†] These authors have contributed equally to this work.

Running head: Plasmonically active portable LSPR sensing chip

* Correspondence: yjcho74@kfri.re.kr (Y. Cho), yunsuk.huh@inha.ac.kr (Y. S. Huh)

Stability and Dispersion of Gold Nanoparticle Solution

After AuNPs reduction with citric acid no further washing was performed. In addition, when the pH of the synthesized gold nanoparticles solution was measured, it was 3.0 – 3.5, and no aggregation of AuNPs occurred after 1 year of synthesis (Figure S1).

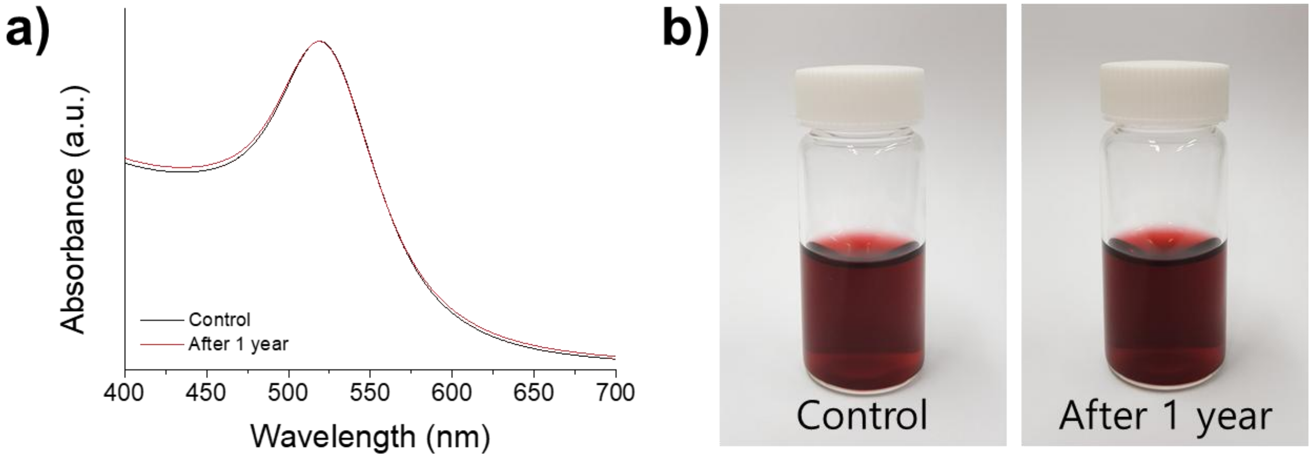

FIGURE S1. Stability and dispersion of gold nanoparticle solution. a) Absorbance of gold nanoparticle solution after synthesis. b) Dispersion image of gold nanoparticle solution.

Preparation of LSPR sensors chip

The LSPR sensor chip was prepared on glass and PC (polycarbonate) substrate [5 cm x 0.8 cm (length x width)]. All the substrates were immersed into methanol and subjected to ultrasonic treatment for 20 min to remove the impurities. The substrates were then washed with distilled water and methanol three times, after which 0.5% APTES as an amine group linker was coated onto the substrates at 60°C for 1 h. The glass and PC substrates were then subsequently washed with distilled water five or six times with to remove any loosely bound APTES, after which the substrates were immersed into AuNPs solution for 16 h. Next, we checked the color change of the amino functionalized surface of glass and PC substrates from colorless to burgundy color, which indicated the adhesion of AuNPs onto the surface of amino functionalized glass and PC substrates.

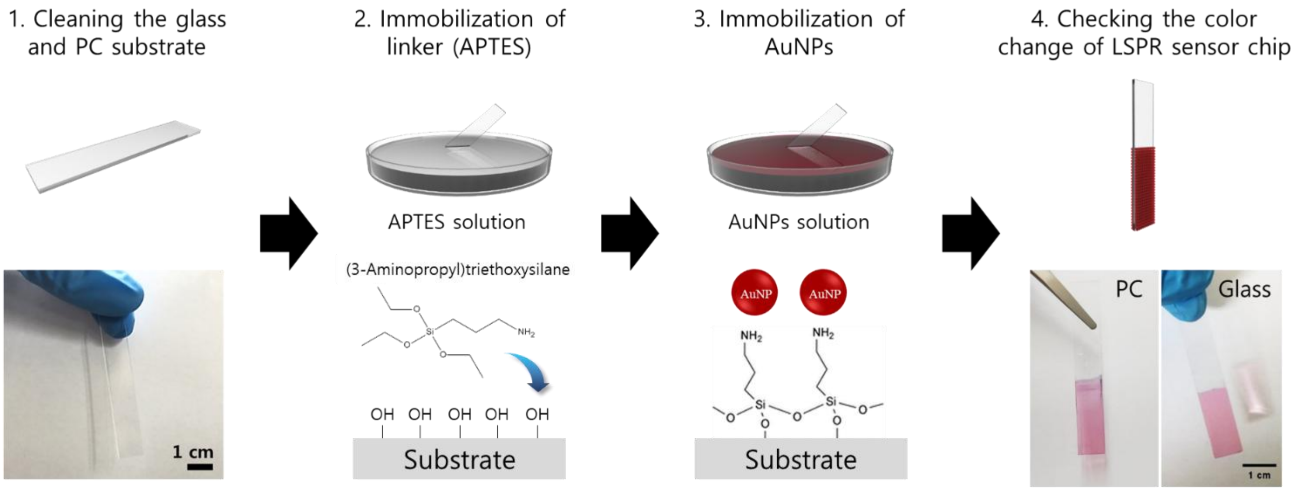

FIGURE S2. Schematic of immobilization of APTES and fabricating LSPR sensor chip.

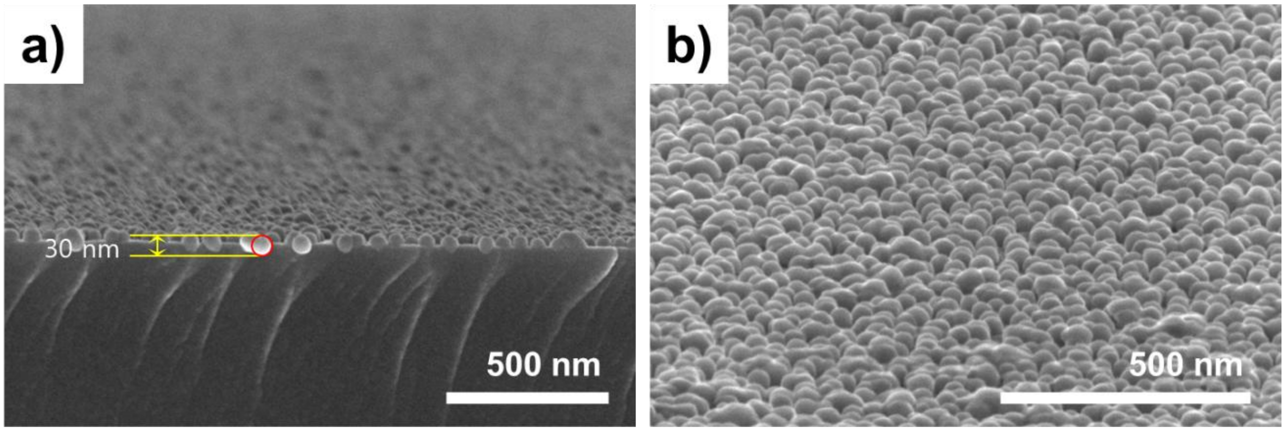

FIGURE S3. (a) SEM tilt image of LSPR sensor chip with single layer of AuNPs, (b) SEM image of LSPR sensor chip surface with single layer of AuNPs.
